# A Unique Surgical Case of Ancient Calcified Intravascular Papillary Endothelial Hyperplasia in the Tibia with Knee Joint Osteoarthritis

**DOI:** 10.1111/os.13495

**Published:** 2022-09-30

**Authors:** Hironori Kitajima, Akihiro Shioya, Rie Kadoguchi, Jia Han, Hinako Sawano, Katsutaka Yonezawa, Yoshiaki Shimizu, Kentaro Hiromura, Mitsuteru Yokoyama, Takayuki Nojima, Sohsuke Yamada

**Affiliations:** ^1^ Department of Orthopaedic Surgery Kanazawa Medical University Himi Municipal Hospital Toyama Japan; ^2^ Department of Pathology and Laboratory Medicine Kanazawa Medical University Ishikawa Japan; ^3^ Department of Pathology Kanazawa Medical University Hospital Ishikawa Japan; ^4^ Department of Pathology Kanazawa Medical University Himi Municipal Hospital Toyama Japan; ^5^ Department of Pathology, Graduate School of Medical Sciences Kanazawa University Ishikawa Japan

**Keywords:** intravascular papillary endothelial hyperplasia, knee osteoarthritis, total knee arthroplasty

## Abstract

**Background:**

Intravascular papillary endothelial hyperplasia (IPEH) is a reactive lesion histopathologically characterized by papillary growth of vascular endothelial cells. IPEH is most commonly found in the skin and subcutaneous tissues of the head, neck, and extremities. Furthermore, it has been reported to occur in oral surgery, but its occurrence in bone is extremely rare.

**Case Presentation:**

We present the case of a 77‐year‐old man with a chief complaint of left knee arthralgia. The knee joint X‐ray showed Kellgren–Lawrence grade 4 osteoarthritis and a mass lesion with decreased permeability within the bone in the medial part of the proximal tibia. Computerized tomography (CT) scan of the left knee showed a localized mass in the left proximal tibia with clear margins and granular internal calcification. The preoperative diagnosis was left knee osteoarthritis and a benign tumor of the left proximal tibia (enchondroma or hemangioma). The patient requested surgical treatment, so left total knee arthroplasty (TKA) and resection of the tumor were performed. The pathology revealed a rare intraosseous IPEH with marked calcification.

**Conclusions:**

Since intraosseous IPEH could not be considered from the clinical findings, the pathological diagnosis was the decisive factor. This report showed the world's first case of intraosseous IPEH with marked calcification. Similar to the calcification of intraosseous hemangiomas, we considered the possibility that, in IPEH, the thrombus may fibrosis and organize in concentric circles, causing necrosis at the center and resulting in calcification. TKA was performed on the degenerative knee joint with IPEH, and a good patient outcome was obtained.

## Graphical abstract

Intravascular papillary endothelial hyperplasia (IPEH) is a reactive lesion histopathologically characterized by papillary growth of vascular endothelial cells. IPEH occurring in bone is extremely rare. We present the case of left knee osteoarthritis and an IPEH of the left proximal tibia.

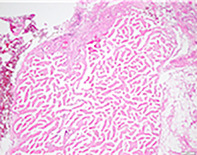

## Introduction

Intravascular papillary endothelial hyperplasia (IPEH) is a reactive lesion histopathologically characterized by papillary hyperplasia of vascular endothelial cells. IPEH was first reported by Masson *et al*. in 1923^1^, and it was named intravascular papillary endothelial hyperplasia by Clearkin and Enzinger in 1976^2^. IPEH is a special type of organizing thrombus in which the vascular endothelial cells proliferate during organizing thrombus process and may occur secondary to hemangioma. The histopathological features of IPEH are as follows: (1) papillary proliferation of vascular endothelial cells is confined to the vessels, (2) most papillary tissue is associated with thrombus formation, (3) vascular endothelial cells are in 1–2 layers and do not show multilayer characteristics, (4) there is little atypia or mitotic figures in vascular endothelial cells, and (5) there is no necrosis of the tissue^3^.

IPEH is most commonly found in the skin and subcutaneous tissues of the head, neck, and extremities. Although there have been scattered reports of IPEH occurring in oral surgery, its occurrence in bone is extremely rare. It has been reported in the mandible, skull, spine, sternum, and tibia as far as we have been able to trace^4^, ^5^, ^6^, ^7^, ^8^. This is the second reported case of IPEH of the tibia and the first isolated case of IPEH of the tibia. In this report, we present a case of old IPEH with associated calcification in the tibia of a patient with knee osteoarthritis.

## Case Report

A 77‐year‐old man presented with a chief complaint of left knee joint pain. He had been aware of left knee joint pain when walking or moving the knee joint for about 15 years. He visited the hospital because of the gradual pain exacerbation and difficulty in walking. He was not aware of any nighttime pain. Five years earlier, he had undergone right total knee arthroplasty (TKA) for right knee osteoarthritis. Additionally, his past medical history included appendicitis, right femoral diaphysis fracture, and early gastric cancer. However, he had no history of hemangioma or trauma to his left knee joint. His family medical history was unremarkable. He had no medications. Physical examination revealed that the patient walked with a single cane and had claudication. There was a medial deformity and tenderness at the medial joint crease in the left knee, and the range of motion was limited to 120° of flexion and −10° of extension.

**Fig. 1:**
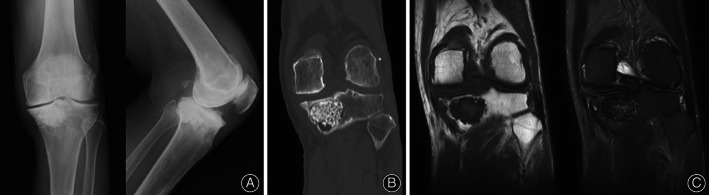
(A) A simple knee joint X‐ray showed degenerative changes in the medial aspect of the left knee, indicating Kellgren–Lawrence grade 4 knee osteoarthritis. There was also a mass lesion with decreased permeability within the proximal tibia bone, but there were no obvious malignant findings such as periosteal reaction. (B) Left knee CT showed a left proximal tibial mass with localized, well‐defined margins and internal granular calcification. (C) Left knee MRI showed a left proximal tibial mass with low‐signal granules and clear margins in both T1 and T2

A simple knee joint X‐ray showed arthropathic changes in the medial aspect of the left knee, indicating Kellgren–Lawrence grade 4 knee osteoarthritis. Additionally, a mass lesion with decreased permeability was observed in the patient's proximal tibia, but there were no obvious malignant findings such as periosteal reaction (Figure 1A). Computerized tomography (CT) scan of the left knee showed a localized mass in the left proximal tibia with clear margins and granular internal calcification (Figure 1B). MRI image of the left knee showed a left proximal tibial mass with low‐signal granules in both T1 and T2 and clear margins (Figure 1C).

Based on these findings, the patient was preoperatively diagnosed with left osteoarthritis of the knee and a benign tumor (enchondroma or hemangioma) of the left proximal tibia. The patient requested surgical treatment, so left TKA and resection of the tumor were performed.

## Surgical Findings

**Fig. 2:**
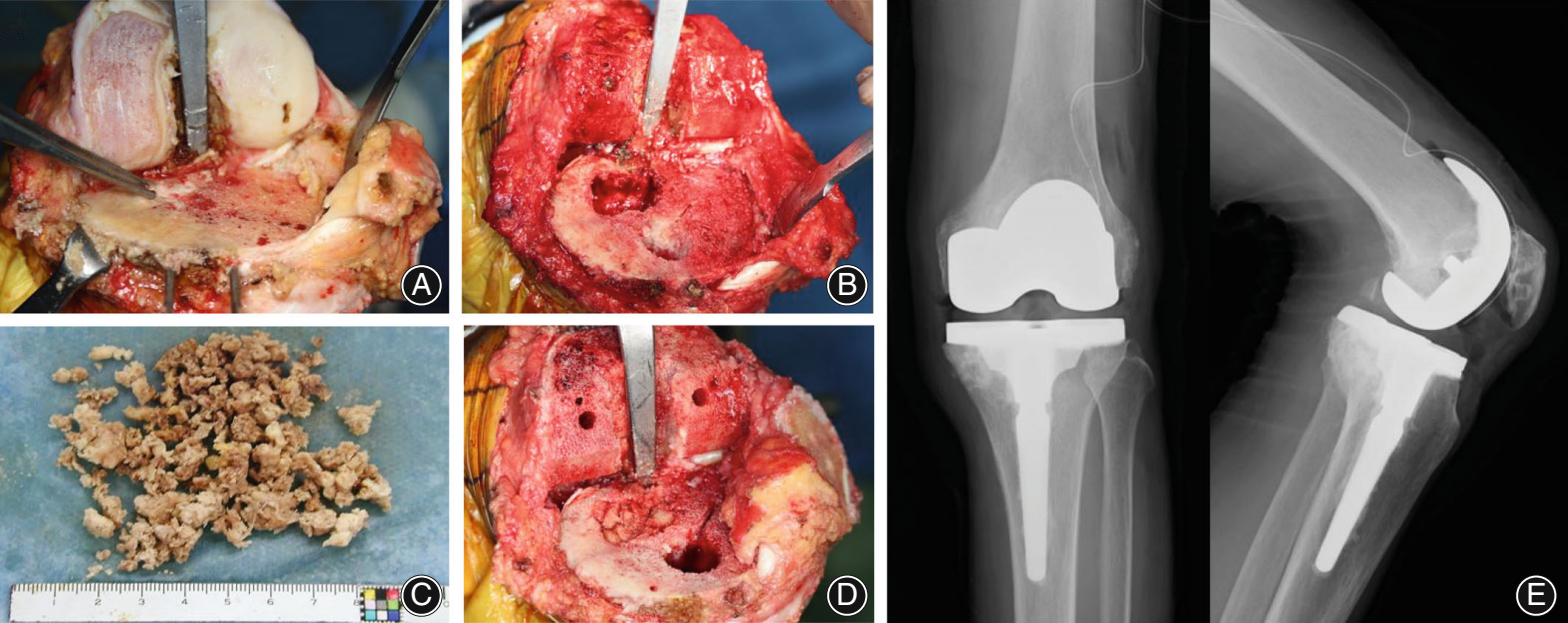
(A) The patient had severe degenerative changes on the medial side of the knee joint surface, and there was no cartilage at all. Tibial facet osteotomy revealed a difference in coloration in the medial‐posterior portion of the tumor. (B) The tumor area in the posterior part of the tibia was scraped with a bone curette. (C) Granular bone‐like tissue was removed from the tumor area. (D) An autologous bone graft was placed from osteotomy bone in the tumor area. (E) An artificial knee joint (Persona® Zimmer Biomet, Warsaw, IN, USA) was placed. A 75‐mm stemmed component was used on the tibial side. The implant was well‐positioned

A median knee incision was made, and the joint surface was exposed through a parapatellar approach. The patient had severe arthropathic changes on the medial aspect of the knee joint, and there was no cartilage at all. Tibial facet osteotomy revealed the tumor of the medioposterior portion of the tibia. The tumor was scraped with a bone curette, and granular bone‐like tissue was removed (Figure 2A–C). The tumor was bleeding slowly but not profusely. The tumor had a limbus and a bony cortex‐like structure. The tumor was scraped and resected with a margin, including that area. To prevent the tumor cells from being pushed into the bone marrow cavity, tumor curettage and removal must be completed before inserting the tibial intramedullary guide. Since the bone defect was confined to the medial condyle and the tibia cortex was preserved, cancellous bone was harvested from the osteotomy bone, and an autogenous bone graft was used to place an artificial knee joint (Persona® Zimmer Biomet, Warsaw, IN, USA). A component with a 75 mm stem was used on the tibial side (Figure 2D,E).

## Pathological Findings

**Fig. 3:**
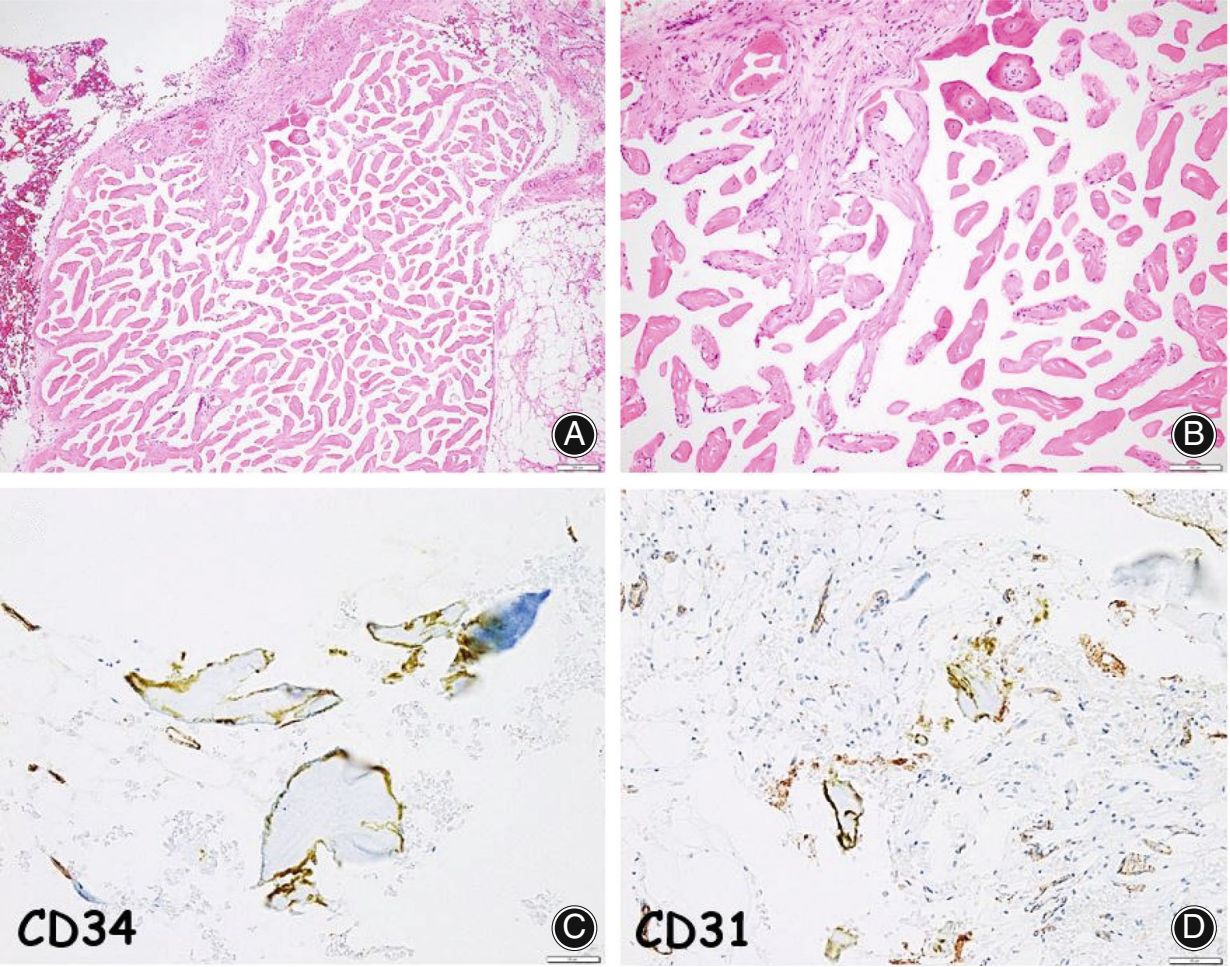
(A) The lesions were predominantly composed of a proliferation of severely degenerative tiny fibrous tissue cores, arranged in a regular and small papillary growth fashion. (B) The mostly denuded lining cells were monolayered, flattened, and bland‐looking. (C and D) Immunohistochemical findings demonstrated that the lining endothelial‐like cells were rarely and specifically positive for CD34 and CD31

Microscopic examination showed that the lesions were predominantly composed of a proliferation of severely degenerative tiny fibrous tissue cores, arranged in a regular and small papillary growth fashion (Figure 3A). The hyalinized and degenerative fibrous components sometimes showed mild and peripheral calcification or ossification. On a high‐power view, the mostly denuded lining cells were monolayered, flattened, and bland‐looking (Figure 3B). Immunohistochemical findings demonstrated that the lining endothelial‐like cells were rarely and specifically positive for CD34 (Figure 3C) and CD31 (Figure 3D), whereas they were negative for cytokeratin and D2‐40 (podoplanin). Our thorough investigation revealed no apparent hemangioma elements, vascular structures, or thrombi. Based on these features, a diagnosis of ancient IPEH in the tibia was ultimately made with unique histological findings.

## Postoperative Course

The implant was well‐positioned. His left knee joint pain improved. The patient was discharged without a cane. The Japanese Orthopaedic Association (JOA) score^9^ improved from 45 to 80. Since the surgery, we have completed approximately a half‐year of routine follow‐up, and the patient remains well without any evidence of tumor recurrence or implant loosening.

## Discussion

IPEH is classified into three subtypes by Hashimoto *et al*.^3^: (1) pure form arising from dilated vessels, (2) mixed form arising from preexisting hemangiomas, and (3) extravascular form derived from blood specimens due to trauma. The previous report of IPEH of the tibia was a case in which microthrombus formation was accelerated by coagulation abnormalities caused by hepatitis C and hepatocellular carcinoma, and IPEH of the tibia was a mixed form of IPEH.^8^ However, in that case, the radiographic findings showed radiolucent lesions of the tibial diaphysis, and the imaging findings were different from those of the present case. Other reports of intraosseous IPEH were often reported as radiolucent lesions. Therefore, there have been no reports of intraosseous IPEH with marked calcification in the mass as in the present case, and it has been difficult to diagnose IPEH clinically.

Calcification has often been reported in IPEH arising from sources other than bone^10^, ^11^. Hemangiomas arising from bone often present with a bony melting image, but sometimes with irregular calcification^12^. Similar to the calcification of hemangiomas, we considered the possibility that, in IPEH, the thrombus may develop fibrosis and organize in concentric circles, causing necrosis at the center and resulting in calcification.

It was impossible to accurately diagnose the subtype pathologically because of the intensity of the degenerative changes in this case. Since there was no obvious history of trauma, we considered the possibility of IPEH in a pure form that had calcified over a long period of time or in a mixed form derived from a hemangioma with calcification. Because of the degeneration and necrosis caused by the chronic mechanical irritation of the knee joint over the years, it was necessary to differentiate angiosarcoma from IPEH based on the relationship with the surrounding tissues and the presence of atypical cells and fission images.

The association between knee osteoarthritis and IPEH has not been described. Because the relationship is unknown, we were unable to prove a causal relationship in this study. We considered the possibility that IPEH in extravascular form may have occurred due to the formation of microvessels in the tibial bone cysts caused by chronic mechanical stimulation. Since there is no previous report on this possibility, we considered that this case of IPEH and knee osteoarthritis was caused by different pathological conditions.

In the general treatment of IPEH, complete resection of the tumor is known to give good results. In this case, tumor resection was performed with sufficient margin^13^. In this case, the bone defect was small at the unilateral condyle, and the bone cortex of the tibia was preserved. Therefore, TKA using an autologous bone graft from osteotomized bone to the scratched part was performed. The 75‐mm stem component of the tibia was used to stabilize the initial fixation because the scraped area of the tumor reached the metaphysis. This stem is suitable for situations where the proximal bone is deficient^14^. Although there have been few reports of IPEH recurrence, there have been reports of recurrence in cases where resection was inadequate, so careful follow‐up is necessary^13^.

### Conclusion

The pathological diagnosis was the decisive factor since intraosseous IPEH could not be considered only from the clinical findings. This report is the world's first report of intraosseous IPEH with marked calcification. Similar to the calcification of intraosseous hemangiomas, we considered the possibility that, in IPEH, the thrombus may develop fibrosis and organize in concentric circles, causing necrosis at the center and resulting in calcification. TKA was performed on the degenerative knee joint with IPEH, and a good patient outcome was obtained.

## Authors' Contributions

HK and SY participated in the conception of the study and writing of the manuscript. HK, KY, YS, KH, and MY performed clinical imaging interpretation and surgery. HK, AS, RK, JH, HS, TN, and SY performed the pathological/immunohistochemical interpretation of this lesion. All authors have read and approved the final manuscript.

## Ethics Approval

Our institution does not require ethical approval for reporting individual cases or case series.

## Informed Consent

Written informed consent was obtained from the patient for their anonymized information to be published in this article.

## Availability of Data and Materials

The dataset supporting the findings and conclusions of this case report is included within the article.

## Competing Interests

The authors declare no conflicts of interest in association with this study.

## Funding

None.
